# Institutional Pressures and Servitization Paradox: The Moderating Effect of Organizational Identity Orientations

**DOI:** 10.3389/fpsyg.2022.901732

**Published:** 2022-06-30

**Authors:** Hui Wang, Xiaojing Lu, Chaping Hu, Haijun Wang

**Affiliations:** ^1^Center for Brand and Advertising Research, School of Media and Communication, Wuhan Textile University, Wuhan, China; ^2^Department of Marketing, School of Business Administration, Jimei University, Xiamen, China; ^3^Department of Marketing, School of Politics and Economic Management, Guizhou Minzu University, Guiyang, China

**Keywords:** manufacturing servatization, institutional pressures, organizational identity orientations, enterprise’s performances, servitization paradox

## Abstract

It is believed in many studies that the servitization of manufactures is driven by internal economy, but the situation in China may be somewhat different. In this study, we consider the influence of external institutional environment on manufacturers’ servitization and the final performance, and discuss the moderating effect of organizational identity orientation on firms’ response to external institutional pressures. We conduct a survey where we collect responses from 312 manufacturers in China. Regression analyses are performed to test whether institutional pressures (normative pressure, mimetic pressure, and coercive pressure) coming from the external institutional environment have a positive effect on the level of manufacturing servitization or not. Moreover, we study if this positive effect is moderated by the individualistic identity orientation and the relational identity orientation. We also examine the impact of servitization strategy on manufacture’s market performance and financial performances. Furthermore, we separate out the influences comes from institutional pressures by using a new estimated method and try to explain the cause of “servitization paradox.” Our study is innovative in that it bridges the servitization and institutional theory, and provides practical guidance for the adoption of manufacturing servitization strategy.

## Introduction

More and more manufacturing enterprises have achieved success through servitization. For example, IBM has transformed itself from a simple hardware manufacturer to an overall solution provider covering hardware, network and software services, and achieved annual profit growth. Rolls Royce is the world’s largest aero-engine manufacturer. It has increased its revenue from services by transforming its business model, expanding its services such as engine maintenance, leasing, data analysis and management, and binding customers to service contracts. Most of its revenue comes from services, and it sells its aero-engines in the form of “rental service hours,” during which time it undertakes all maintenance, repairs and services (Johnstone et al., 2009). The servitization strategy in manufacturing industry can solve the competitive dilemma, such as serious product homogeneity, lack of competitive advantage of enterprises, product profit margin decline and customer demand heterogeneity, and bring sustainable competitive advantage to traditional manufacturing industry. Therefore, it has attracted more and more attentions from researchers (Iriarte et al., 2018; Zhou et al., 2020; Khanra et al., 2021; Rabetino et al., 2021).

Previous studies have argued that the servitization of manufacturing enterprises is driven by internal economic benefits. For example, enterprises conduct servitization in order to obtain product differentiation, market competitive advantage, and customer loyalty (Gebauer and Fleisch, 2007; Gebauer et al., 2011). Other studies suggest that the servitization of manufacturing enterprises is driven by external environmental pressure, including customer demand diversity, market complexity, market competition intensity and environmental effect (Ren and Gregory, 2007; Roy and Cheruvu, 2009; Turunen and Finne, 2014). However, the influence of external institutional pressure on the servitization of manufacturing enterprises is still to be elucidated from the perspective of institutional environment. In the past, some researches believed that institutional pressure would affect the innovation of enterprises, and the servitization of manufacturing enterprises is also an innovation activity, which is in the aspects of product-service portfolio, interaction interface and value transmission (Heugens and Lander, 2009). The servitization of manufacturing enterprises is exactly to transform the traditional consumption pattern, value creation mode and resource utilization mode through the service system (Kang and Wimmer, 2008; Baines et al., 2009; Martinez et al., 2010). Therefore, this study intends to explore how manufacturing enterprises make servitization strategic decisions when faced with the pressure of external institutional environment.

Servitization in manufacturing refers to the transformation from product-led logic to service-led logic. Servitization is to add value to core products and provide customers with a more complete product-service portfolio, including tangible goods, services, support, self-service and knowledge (Vandermerwe and Rada, 1988). In the past, it was believed that the choice of manufacturing servitization strategy was driven by internal economic benefits. However, we believed that institutional pressure provided a non-economic explanation for manufacturing servitization strategy. Enterprises located in organization field consist of suppliers, customers, competitors and government agencies (DiMaggio and Powell, 1983), and inevitably influenced by the institutional environment such as social rules, norms, rules or culture, this influence is the institutional pressure, which will affect organizational form, structure or behavior to be either reasonable, acceptable and supportable or not, regardless of whether these forms or behaviors contribute to the operational efficiency of the organization (Qian and Burritt, 2008). Due to the change of customer demand and ecological benefits, the servitization strategy of manufacturer may be affected by institutional pressure. Therefore, this study raises the following research questions: will institutional pressure affect manufacturers’ servitization strategy?

In addition, enterprises under institutional pressure will not blindly follow, but strategically respond to such pressure (Heugens and Lander, 2009). Previous studies have found that organizational culture can moderate the relationship between institutional pressure and the adoption of electronic supply chain systems (Liu et al., 2010). It is believe that if a company has strong capabilities, it will reduce its dependence on external environment and have more say in its own business strategy (Li and Ding, 2013). At the same time, they have more confidence in controlling risks and dealing with environmental uncertainties, which will reduce the imitating behavior of peer companies’ strategies. All these studies explore the positive response of organizations to institutional pressure from the internal perspective of enterprises. By introducing the concept of organizational identity orientation, this study explains that enterprises with different organizational identity orientation may have different servitization strategy choices when faced with institutional pressure, that is, what kind of enterprises will be more affected by the “servitization paradox.” We believe that institutional pressure is the influence of external stakeholders on an organization’s behavior through social norms and institutional expectations, and how an organization views itself and the relationship between itself and external stakeholders (i.e., organizational identity orientation) will lead to different responses to institutional pressure. Therefore, this study raises the following research questions: will organizational identity orientation affect the relationship between institutional pressure and manufacturer servitization?

Finally, past studies have different conclusions on the impact of servitization on enterprise performance (Zhou et al., 2020), and a large number of studies believe that servitization will positively affect enterprise performance (Eggert et al., 2011; Kastalli et al., 2013), but there are also quite a few studies confirming that the servitization transformation of manufacturing companies will harm the performance of manufacturing companies (Neely, 2008; Visnjic et al., 2012; Suarez et al., 2013; Marjanovic et al., 2020). Others believe that there is a nonlinear relationship between servitization and enterprise performance (Fang et al., 2008; Kohtamäki et al., 2013; Zhou et al., 2020). This study believes that how an enterprise responds to external institutional pressures, whether it responds actively or passively, will affect its servitization related strategic decisions, and the difference of strategic decisions will affect subsequent performance. Therefore, this research raises the third research question: How does the servitization strategy of a manufacturing company affect enterprise performance? In particular, what is the impact of servitization on enterprise performance when enterprises succumb to institutional pressure?

Through the study of the above three issues, this study specifically analyzes the non-economic factors driving the servitization strategy of manufacturers, namely the impact of institutional pressure, and specifically analyzes the boundary conditions of institutional pressure’s impact on enterprise strategy, making several contributions to the expansion of institutional theory. First, we promote the application of institutional pressure. In the past, the influence of institutional pressure on enterprises mainly focuses on the influence of corporate social responsibility behavior and moral behavior (Martin et al., 2011). Several researches focus on the innovative adoption of enterprises, such as information technology, electronic channel system, electronic supply chain management system and total quality management system (Teo et al., 2003; Liang et al., 2007; Liu et al., 2010). Few researches focus on the institutional drivers of manufacturing servitization strategy, which can help us understand the external institutional environment of manufacturing servitization strategy. Secondly, we study the boundary conditions of the influence of institutional pressure on the servitization strategy of enterprises from a new perspective. In the past, the results of studying the effect of institutional factors on the innovation decision-making of enterprises are chaotic. Some studies have found that the influence of coercive pressure is significant (Teo et al., 2003), while some studies have found that it is not significant (Liang et al., 2007). In view of this, more studies are needed to explore the potential moderating variables in the process of enterprises coping with institutional pressure. To date, it is studied the moderating effect of social identity on the relationship between institutional pressure and audit quality (Wang et al., 2017), and another research studied the moderating effect of organizational culture by combining institutional theory and organizational culture theory (Liu et al., 2010). Therefore, it can be seen that there is lack of research on the boundary conditions of the impact of institutional pressure on the servitization of enterprises. The introduction to the concept of organizational identity orientation in this study is helpful to increase the understanding of the process of enterprises’ response to institutional pressure.

## Literature Review and Theory Hypotheses

### Servitization Strategy of Manufacturing Industry

The servitization of manufacturing industry refers to the transformation from product-led logic to service-led logic (Vandermerwe and Rada, 1988). Previous studies have classified manufacturing service delivery types into: service delivery supporting customer products and service delivery supporting customer behavior (Mathieu, 2001; Gebauer and Friedli, 2005). The service delivery supporting customer products refers to the relevant services provided by product supplier enterprises for customers in order to ensure that the products purchased by customers play the appropriate functions or facilitate customers to manipulate all products, including product maintenance, technology upgrade, operation monitoring, commissioning and installation, technical transformation. Service delivery supporting customer behavior refers to relevant services provided to customers by product suppliers aiming at exploring how to support specific customers’ innovation activities and core organizational capabilities to build or promote the realization of customers’ organizational mission or goals, including product research and development support, business process optimization, business consulting services, professional support for production system, new project establishment support (Gebauer and Friedli, 2005).

There are many researches on servitization strategy of manufacturing industry, most of which focus on the driving force of servitization strategy selection of manufacturing industry. For example, customer demand diversity, market complexity, market innovation, competitive intensity and resource availability have important influences on driving manufacturing enterprises to expand service business (Gebauer, 2008; Turunen and Finne, 2014). Some studies also focus on the influence of servitization strategy of manufacturing industry on manufacturers’ performance. Most scholars believe that servitization strategy is helpful to improve the business performance of enterprises (Gebauer and Friedli, 2005; Antioco et al., 2008; Visnjic et al., 2012). Some other scholars do not believe that servitization always have a positive effect on enterprise performance. They put forward the “servitization paradox” and believe that servitization may cause the loss of market value and profits (Mathieu, 2001; Neely, 2008; Kastalli et al., 2013). We pay attention to the influence of institutional environment on the servitization strategy of manufacturers and the research on boundary conditions may help to understand this paradox.

### Institutional Pressure and Servitization Strategy of Manufacturing Industry

Institutional pressure refers to the influence of shared norms and values in the external institutional environment on organizational form, structure and behavior, and is considered as a factor that can promote an enterprise to make a certain strategy. Institutional theory holds that institutional environment provides social expectations and norms for appropriate organizational structure, operation, behavior and practice (DiMaggio and Powell, 1983). Compliance with these expectations and norms is very important for enterprises to maintain their legitimacy, which ensures enterprises to obtain important scarce resources (DiMaggio and Powell, 1983; Heugens and Lander, 2009; Huo et al., 2013). Therefore, in order to obtain legitimacy and support from the environment, organizations tend to comply with the pressure from the external institutional environment and comply with various norms and systems recognized by important stakeholders in a particular organizational field.

Some studies have shown that institutional pressure can affect enterprises’ decision-making. It believes that enterprises may adopt electronic supply chain management system in response to institutional pressure from business environment (e.g., customers, suppliers and industry) (Liu et al., 2010). It is confirmed that coercive pressure from manufacturers will affect the implementation and adoption of suppliers’ business management strategies (Rogers et al., 2007). Other studies have also confirmed that the diffusion of total quality management (TQM) principles and practices is also through mimetic mechanism. Early adopters are driven by economic benefits, but late adopters adopt TQM for rationality (Westphal et al., 1996). It is possible that mimetic behavior may not necessarily bring economic benefits, but enterprises will still yield to the pressure of mimetic to reduce risk perception and pursue rationality (Liu et al., 2010). Mostly, institutional theory is used to provide explanations for whether or not to take an innovation or the intention to take an innovation (Teo et al., 2003). In the context of supply chain management (SCM), scholars adopt internet-enabled and IT-enabled SCM by institutional theory testing (Teo et al., 2003; Liu et al., 2010). In many cases, the servitization strategy of manufacturing industry is also a service innovation strategy, such as adopting new technologies to improve and change existing service processes and service products. We believe that institutional pressure provides an institutional explanation for the servitization strategy of manufacturing industry.

The dimension of institutional pressure adopted in this paper, which can be divided into normative pressure, mimetic pressure and coercive pressure (DiMaggio and Powell, 1983). In the context of servitization of manufacturing industry, the mimetic pressure mainly comes from the perception of the success of enterprises’ competitors in adopting servitization strategy. Coercive pressure refers to the degree to which the main target customers demand the service of the manufacturer. Normative pressure comes from partners, competitors, and other companies in the industry adopt service delivery. Next we discuss the impact of three institutional pressures on manufacturers’ servitization strategies.

#### Normative Pressure

**Figure 1:**
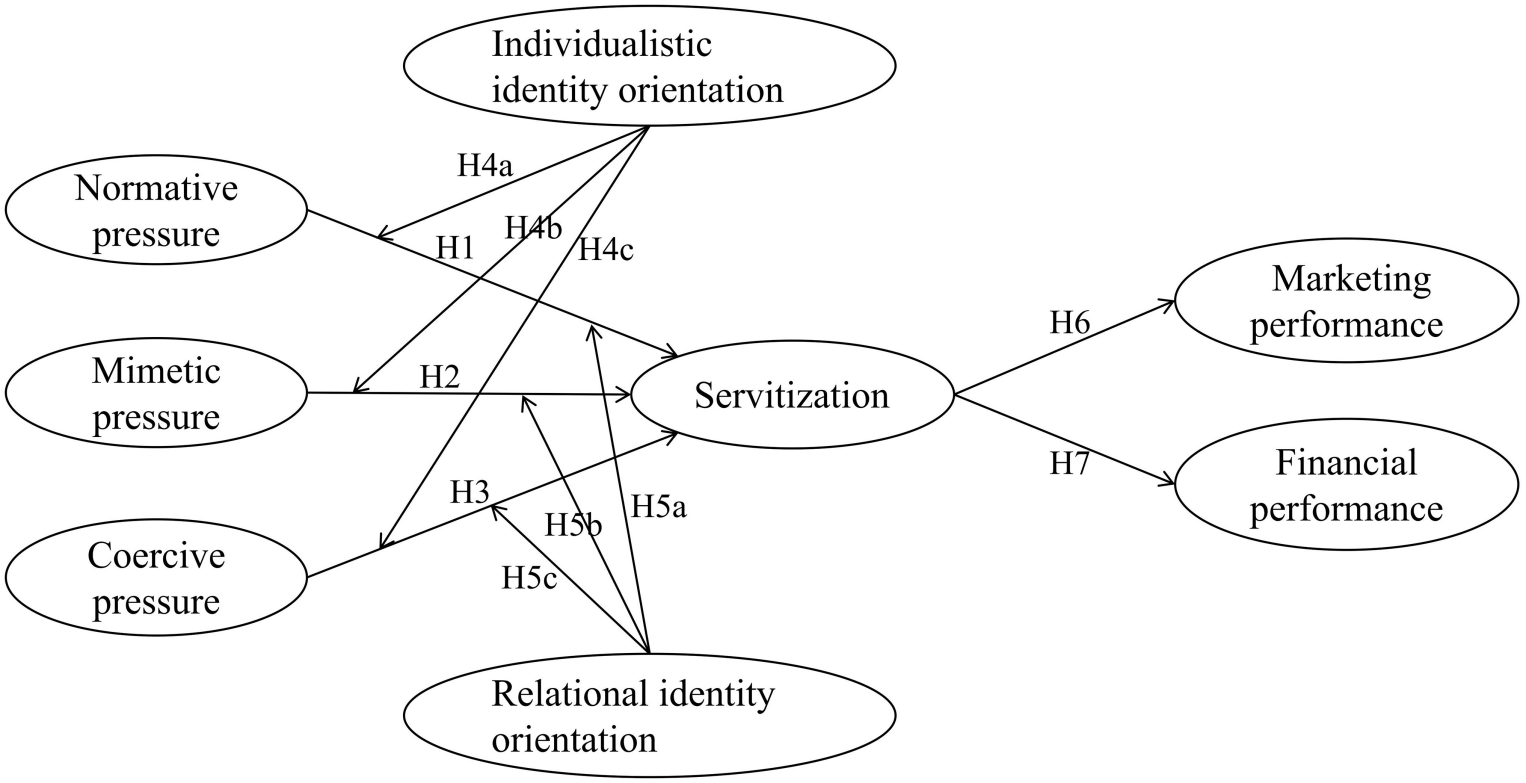
Theoretical models and hypotheses.

The prevalence of an innovation in the context of an organization exerts normative pressure on companies to adopt the innovation (Liu et al., 2010). But sometimes, normative pressure comes from shared paradigms and values, which has been accepted in this field. Companies are compelled to seek legitimacy by these shared norms and values. Under the current research background, if suppliers and partners increase investment in manufacturers’ services, the interpersonal contact and emotional embedding of service provision will enhance the relationship between each other and promote the establishment of trust to create more marketing opportunities and cooperation opportunities for enterprises (Gebauer and Fleisch, 2007). If competitors and partners widely accept and support service delivery, manufacturers will perceive the normative pressure. If manufacturers do not adopt service delivery, they may be isolated. In order to obtain key resources, companies tend to conform to the normative pressure, increase the investment on the systems and services, transform from only providing products to provide products and services. Also, shared norms and values promote the involvement of manufacturers, upstream suppliers and downstream buyers in response to the normative pressure, manufacturers will upward get into the stakeholder network and will also be involved in the product development, design, production processes of both upstream suppliers the downstream buyers to maintain rationality and obtain scarce resources in the supply chain. Therefore, we propose the following hypothesis (Figure 1):

*H1*: The perceived normative pressure by the manufacturer positively affects the manufacturer to adopt servitization strategy.

#### Mimetic Pressure

In the servitization strategy of manufacturing industry, when manufacturers learn how competitors gain competitive advantages and good corporate performance from the servitization strategy, they will perceive the mimetic pressure and thus mimetic these successful competitors (Mitra and Singhal, 2008). In fact, the servitization strategy of manufacturers is full of uncertainty and risks, and there is a “service paradox” (Kastalli et al., 2013). In order to improve their service level, many manufacturers invest a lot of money, which not only gains the corresponding income, but also causes the loss of market value and profit (Gebauer and Friedli, 2005). Therefore, in the context of uncertainty and risk, it is a rational choice for manufacturers to decide whether or not and to what extent to adopt servitization strategy according to their competitors’ service delivery performance.

In addition, servitization can create customer dependence by building barriers against competitors, third parties and customers (Vandermerwe and Rada, 1988). It shows that personalization of value-added services provided by manufacturers will create customer loyalty, as well as high dependence of customers on product suppliers (Corrêa et al., 2007). Moreover, it is pointed out that service-oriented strategy can improve product differentiation and form certain barriers, so as to obtain sustainable competitiveness (Gebauer and Friedli, 2005). Firms feel that their market position is threatened and even firms’ future existence is threatened when they perceive that their major competitors have adopted servitization strategies and acquired differentiated competitive advantages and customer loyalty (Lieberman and Asaba, 2006). Such threat perception also provides a reasonable explanation for the Assimilation of enterprise systems: the effect of institutional power and the mediating role of top management when making decisions that mimic those of successful competitors (Liang et al., 2007). Therefore, we propose the following hypothesis (Figure 1):

*H2*: The mimetic pressure perceived by the manufacturer positively affects the servitization strategy of the manufacturer.

#### Coercive Pressure

The coercive pressure is carried out through the relationship channels among members in the network (Teo et al., 2003), which is mainly derived from the demand of enterprise target customers for product and service provision. The servitization strategy of manufacturers itself is a customer-oriented strategy, which emphasizes that enterprises should meet the needs of customers to the maximum extent (Mathieu, 2001). Research shows that customers are increasingly inclined to buy products that provide integrated problem solutions such as installation, training, repair and maintenance, upgrade and transformation (Gebauer and Fleisch, 2007). Therefore, manufacturers should not only provide high-quality tangible products, but also provide more targeted package solutions. Therefore, when a manufacturer perceives a buyer’s need for service delivery, the manufacturer will comply with this requirement in order to avoid losing orders and gain access to scarce resources. Therefore, we propose the following hypothesis (Figure 1):

*H3*: The coercive pressure perceived by the manufacturer positively affects the servitization strategy of the manufacturer.

### Moderating Effect of Organizational Identity Orientation

We believe that organizations in making the strategy of service from the external environment of the institutional pressure (normative pressure, mimetic pressure, and coercive pressure), but we also recognize that organizations do not passively submit to all the system pressure and reconcile their interests with pressures (Oliver, 1991). As a result, the response of organizations to the institutional pressure should be different, depending on their own characteristics.

In the past, it was believed that the response of an organization to the pressure of external environment would be restricted by its own resources and capabilities (Heugens and Lander, 2009; Li and Ding, 2013). In fact, how does an organization view its relationship with surrounding organizations determine how it behaves. The theory of organization identity orientation provides a framework for us to explain the relationship between organizations and other organizations especially stakeholders. It views the relationship between itself and stakeholders from three perspectives, namely, it regards itself as an independent individual, a partner of stakeholders, or a member of a larger collective. These three perspectives reflect the three categories of organization identity orientation, namely, individualistic identity orientation, relational identity orientation, and collectivistic identity orientation) (Brickson, 2005). The three types of organizational identity orientation differ in the perspective of self-definition, basic motivation of interaction with stakeholders, and reference frame of self-evaluation. Therefore, this paper proposes a new perspective that organizational identity orientation as the perception of the relationship between organizations and stakeholders will moderate the influence of institutional pressure on organizational behavior.

In this study, we only investigate the moderating effect of individualistic identity orientation and relational identity orientation. Because collectivistic identity orientation is often seen in nonprofit organizations (Brickson, 2007) and in the pre-survey, we cannot find big enough manufactures with collectivistic identity orientation.

#### Moderating Effect of Individualistic Identity Orientation

Enterprises with the individualistic identity orientation pay more attention to their own interests in the process of interacting with stakeholders. Generally, it pursues its own goals when maintaining a relationship with stakeholders, such as profit maximization (Brickson, 2005). It believes that relationship maintenance is instrumental, and in order to maintain its own uniqueness, the relationship between the organization and stakeholder groups is weak and changeable, that is to say, in order to ensure efficiency, the organization can completely change partners (Brickson, 2007). Therefore, the relationship between organizations with individualistic identity orientation and stakeholders is characterized by “instrumentality” and “weak connection.”

Therefore, when faced with normative pressure from external environment with shared norms and values and the mimetic pressure from competitors and partners, manufacturers with individualistic identity orientation may pay more attentions to their own interests and consider the risks and benefits of servitization. Therefore, individualistic identity orientation can weaken the positive influence of normative pressure and mimetic pressure on servitization strategy. Therefore, we propose the following hypotheses (Figure 1):

*H4a*: Individualistic identity orientation negatively moderates the impact of normative pressure on manufacturer servitization.

*H4b*: Individualistic identity orientation negatively moderates the impact of mimetic pressure on manufacturer servitization.

In addition, when faced with coercive pressure from customers to demand services, we believe that manufacturers with individualistic identity orientation will succumb to such pressure to provide services. Manufacturers perceive that the needs of customers change with the development of economy, and they need to provide more services other than products to meet their needs. If manufacturers do not provide services, they will face the loss of competitive advantage and market position. Then, for the sake of profit, the manufacturer will satisfy the customer’s service demand. Therefore, we propose the following hypothesis (Figure 1):

*H4c*: Individualistic identity orientation positively moderates the impact of coercive pressure on manufacturer servitization.

#### Moderating Effect of Relational Identity Orientation

An organization with relational identity orientation defines its social self from the perspective of partnership and considers itself as a partner of the stakeholder group (Brickson, 2005). The relationships between itself and stakeholder groups form based on the attention and trust and the connections between itself and stakeholder groups in pairs make up all the meaning of organization. The organizations deem the relationship maintenance between itself and stakeholder groups as its main goal, rather than as a means to other purposes, sincerely consider the interests of the stakeholder groups. Therefore, the relationship between organizations with relational identity orientation and stakeholder groups is characterized by “mutual concern and trust” and “strong connection.”

Therefore, when faced with normative pressure from professional environments and mimetic pressure from competitors and collaborators, we believe that manufacturers with relational identity orientation will conform to normative pressure and mimetic pressure. Because manufacturers with relational identity orientation tend to build relationships of mutual concern and trust with other organizations in the overall professional environment, and shared values and norms also facilitate the formation of trust relationships and strong connections. This relationship quality causes the manufacturer to comply with the normative pressure and the mimetic pressure and obtain the rationality of the interaction with stakeholders. In addition, due to good relations with other organizations in the professional environment, such as suppliers, competitors and partners, manufacturers have a clearer understanding of the process of their servitization strategies, which will reduce their perceptions of the uncertainty of servitization strategy and enhance their confidence in the success of servitization strategy. Therefore, we propose the following hypothesis (Figure 1):

*H5a*: Relationship identity orientation positively moderates the impact of normative pressure on manufacturer servitization.

*H5b*: Relational identity orientation positively moderates the impact of mimetic pressure on manufacturer servitization.

In addition, when faced with coercive pressure from customers, we believe that manufacturers with relational identity orientation may not succumb to such pressure. Since manufacturers with relational identity orientation and customers tend to establish a good relationship of trust and sincerely consider the interests of the other side. When the manufacturers find changes in customer needs, they may solve them in other ways. Because manufacturers with relational identity orientation tend to have good relationships with all stakeholders in the external environment, they may not always be able to meet everyone’s needs when dealing with these complex and conflicting stakeholder needs. Therefore, we propose the following hypothesis (Figure 1):

*H5c*: Relational identity orientation negatively moderates the impact of coercive pressure on manufacturer servitization.

### Servitization Strategy and Performance of Manufacturers

#### Servitization Strategy and Marketing Performance

First of all, services are provided to customers by front-line employees of the enterprise, therefore, the servitization strategy can promote the development of the relationship between employees and customers, and help the enterprise and customers to establish a long-term and lasting relationship of mutual trust (Heide and John, 1990). This kind of lasting and good relationship brought by the service is likely to promote the repeated purchase of the service, make the customers depend on the enterprise, and improve customer loyalty (Corrêa et al., 2007). At the same time, services can also strengthen the opportunity for enterprises to contact with customers, thus improving the opportunities for customers to purchase other products or services of enterprises (Mathieu, 2001; Malleret, 2006).

Secondly, it is showed that with the development of economy, customers’ demands changed from product-centered to product-centered service or utility (Oliva and Kallenberg, 2003). Studies show that when customers make purchasing decisions, whether the core products provided by enterprises contain service components or not will become an important factor influencing consumer decisions (Mathieu, 2001; Gebauer and Fleisch, 2007). Therefore, manufacturing enterprises can provide customized problem solutions more in line with customers’ expectations, so as to better meet customers’ personalized needs, improve customer satisfaction, and make customers become more dependent on their own enterprises.

Finally, employees of manufacturing enterprises can gain important knowledge and information about customer preferences and demands through in-depth understanding of customer needs through extensive service contact with customers. Enterprises can use this information to improve products and services, produce and sell products according to customers’ personalized needs, and thus create more value for customers (Berry and Parasuraman, 1997). The increase in customers’ purchase intention and satisfaction degree will improve the marketing performance of enterprises. Therefore, we propose the following hypothesis (Figure 1):

*H6*: The servitization strategy of manufacturers positively affects the marketing performance of enterprises.

#### Servitization Strategy and Financial Performance

First, the servitization strategy requires manufacturers to invest in special service resources and capabilities to deliver services smoothly. However, the service profit model is quite different from the one-time product sales profit model, and these considerable investments in the service will temporarily reduce the corporate profit margin (Gebauer and Friedli, 2005). Study also confirmed through empirical research that only when the service activities of manufacturers accumulate to realize the scale economy of service they can deliver more cost-effective service, and then the profit margin of manufacturers will increase (Kastalli et al., 2013). However, a large-scale data survey on 10,028 manufacturing enterprises was conducted and found that only 21% of the manufacturing enterprises were successful in servitization, which means that most of the enterprises did not reach the scale economy of service but were in the stage of low profit margin for a long time (Neely, 2008).

Second, services and product manufacturing businesses require different organizational processes, cultures, leadership and structures (Deshpandé et al., 1993; Vargo et al., 2008). Servitization strategy means that the enterprise should integrate a mixture of organizational elements (process, culture, and so on) into the same entity. Therefore, service transformation may cause internal confusion, tension and even outright conflict (Fang et al., 2008). Such conflicts within an organization will reduce the enthusiasm and efforts of employees, destroy resource utilization rate and productivity, lead to second-best resource deployment and allocation decisions, and thus weaken the ability of enterprises to create value. Meanwhile, in order to transform manufacturing enterprises from product-centered to service-oriented organization (Baines et al., 2009; Alghisi and Saccani, 2015), the organizational structure must be reconstructed to be consistent with the business strategy (Gebauer et al., 2010). However, the organizational structure is inertial. Breaking or reformulating the rules of the game is bound to affect the vested interests of some internal people and cause internal political costs (Mathieu, 2001). From a financial perspective, the changing organizational structure will increase the financial needs in the initial stage of servitization, such as the development of new functional groups and the recruitment of new human resources (Neely, 2008; Parida et al., 2014). Therefore, bankruptcy is possible if the expected returns are not achieved within a specified period of time (Benedettini et al., 2015, 2017).

Finally, enterprises usually operate under resource constraints, and adopting service transformation strategy may sacrifice the resource input level of their core products and manufacturing capacity (Bourgeois, 1981). In other words, the combined resource requirements for core product activities (e.g., R&D and manufacturing improvement) and service activities may dilute enterprise resources, so that neither type of activity has sufficient resources to achieve success. Therefore, the deployment of corporate resources between existing businesses and new businesses that require new skills, capabilities and competitiveness should have a negative impact on a company’s performance and ultimately its market valuation, at least in the short term. These negative effects are likely to persist until companies develop the core competencies needed to compete effectively for new businesses and managers learn how to optimize the allocation of resources in different areas. Therefore, we propose the following hypothesis (Figure 1):

*H7*: The servitization strategy of manufacturers negatively affects the financial performance of enterprises.

## Materials and Methods

### Data Collection and Analysis

For this study, we choose a relatively low degree of vertical integration in the industry such as general equipment manufacturing industry, special equipment manufacturing, electrical machinery and equipment manufacturing, transportation equipment manufacturing, automobile manufacturing and so on, because the upstream and downstream of the value chain in the industry are all manufacturing enterprises (Fan et al., 2009). In this study, the main sampling range is Guangdong, Zhejiang, Jiangsu, Shandong, Hunan, Henan provinces, because the manufacturing enterprises in these provinces are relatively concentrated, and the degree of service is relatively high. Such samples are helpful for us to investigate and understand the drivers and performance of servitization strategy in manufacturing industry.

This study mainly adopts the following methods to collect questionnaires: (1) Asking if there are managers of manufacturing enterprises in Executive Master of Business Administration (EMBA) or Master of Business Administration (MBA) courses of Wuhan University, asking them to help fill in the questionnaires, and giving small gifts as a token of appreciation. (2) Sending questionnaires to the personnel of relevant manufacturing enterprises by email and WeChat through various social relations such as university, graduate students, teachers and friends. (3) According to the introduction of various social relations, we contacted the managers of manufacturing parks in various places and personally sent questionnaires to the managers of enterprises in the manufacturing parks. Through these methods, a total of 1,108 questionnaires were distributed in this study, and 312 valid questionnaires were collected at the end, with a recovery rate of 28.2%. Among these subjects, 7 were senior managers, accounting for 2.2%; there are 54 middle managers, accounting for 17.3%; there were 189 grass-roots managers, accounting for 60.6 percent; the remaining 62 employees are ordinary employees, accounting for 19.9%. 32 of these subjects have served in the company for less than 1 year, accounting for 10.3%. In 1–3 years, there were 179, accounting for 57.4%. From 3 to 10 years, there were 88, accounting for 28.2%. Over 10 years, there were 13, accounting for 4.2%. The distribution of the reliable samples are summarized in Table 1.

**Table 1:** Detailed information of subjects collected in this study.

Basic characteristics	Classification	Number of samples (*n* = 312)	Percentage (%)
Firm age	Less than 3 years	58	18.5%
	3 to 10 years	193	61.9%
	More than ten years	61	19.5%
Firm size	1–500	167	53.5%
	500–1,000	96	30.8%
	More than 1,000	49	15.7%
Nature of enterprise ownership	State-owned and state-controlled enterprises	47	15.1%
	Joint-stock enterprises	55	17.6%
	Private enterprises	154	49.4%
	Foreign capital enterprises	56	17.9%
Industry of enterprise	General equipment manufacturing	18	5.8%
	Special equipment manufacturing	20	6.4%
	Electrical machinery and equipment manufacturing	13	4.2%
	Transportation equipment manufacturing	44	14.1%
	Automobile manufacturing	20	6.4%
	Electronic equipment manufacturing	43	13.8%
	Instrument manufacturing	34	10.9%
	Manufacturing of chemical raw materials and chemical products	39	12.5%
	Nonferrous metal smelting and rolling processing industry	35	11.2%
	Nonmetallic mineral products industry	18	5.8%
	Metal products industry	25	8.0%
	Other	3	1.0%
Province	Guangdong	101	32.4%
	Zhejiang	40	12.8%
	Jiangsu	52	16.7%
	Shandong	51	16.3%
	Hunan	36	11.5%
	Henan	29	9.3%
	Other	3	1.3%

### Measurement

The four major constructs involved in this study were relatively mature concepts that covered a lot of measurement methods in the previous research (DiMaggio and Powell, 1983; Mathieu, 2001; Gebauer and Friedli, 2005; Lieberman and Asaba, 2006; Corrêa et al., 2007; Liang et al., 2007; Alghisi and Saccani, 2015). In order to use these scales in the Chinese environment and truly reflect the characteristics of Chinese manufacturing enterprises, we consulted relevant scholars and entrepreneurs and made modifications and adjustments according to expert opinions.

We adopted the methods of previous studies to measure the servitization strategy of manufacturers (Gebauer and Friedli, 2005; Antioco et al., 2008; Parida et al., 2014), which was divided into 5 items to promote product service and 5 items to promote customer behavior, forming a total of 10 items. Institutional pressure was measured with reference to previous studies (Liu et al., 2010; Huo et al., 2013). The normative pressure in the context of this study mainly comes from suppliers, partners and other enterprises in the same industry that adopt the servitization strategy. The mimetic pressure mainly comes from manufacturer’s competitors that have adopted servitization strategies. The coercive pressure mainly comes from the customer’s demands for the service from manufacturer. For each pressure measurement, 3 items were used. Organizational identity measurement use items from previous studies (Brickson, 2005, 2007). Corporate performance is divided into financial performance and market performance and 3 items are used for reference to measure financial performance and market performance, respectively (Antioco et al., 2008). All dependent and independent variables were measured using a seven-point Likert scale.

Relevant studies show that age, size, ownership, competition intensity, and environmental dynamics have an impact on the adoption of servitization strategy by manufacturers. Although these factors are not the focus of this study, they may affect the results of data analysis to some extent. Therefore, in this study, we controlled for these variables. The enterprise age is the operation years of the enterprise, divided into three stages (1 = less than 3 years, 2 = 3–10 years, 3 = more than 10 years). The number of employees in the enterprise is also divided into three categories (1 = less than 500 employees, 2 = 500–1,000 employees, 3 = more than 1,000 employees). The enterprise ownership model uses state-owned, joint-stock, private, foreign-funded enterprises and others (Nature of Company 1 = state-owned and state-holding enterprises, Nature of Company 2 = joint-stock enterprises, Nature of Company 3 = private enterprises, Nature of Company 4 = foreign-funded enterprises). Competitive intensity uses the level of competition between the same industry known by the enterprise sense (1 = highly competitive column, 0 = not intensely competitive). Environmental dynamics is an enterprise’s perception of the change in market demand of its major customers (1 = fast change, 0 = slow change).

### Descriptive Statistical Analysis

This study first carries out descriptive statistical analysis of all the variables involved in the sample, including servitization, normative pressure, mimetic pressure, coercive pressure, firm age, firm size, environmental dynamics, competitive intensity, enterprise market performance and enterprise financial performance. Specific values include mean value, standard deviation, as shown in Table 2.

**Table 2:** Descriptive statistics and Person correlation matrix.

Variables	Mean	SD	1	2	3	4	5	6	7	8	9	10	11
Servitization	3.560	0.898	–										
Normative pressure	3.488	1.062	0.233^***^	–	–	–	–	–	–	–	–	–	–
Mimetic pressure	3.359	1.140	0.300^***^	0.081	–	–	–	–	–	–	–	–	–
Coercive pressure	3.398	1.132	0.248^***^	0.204^***^	0.028	–	–	–	–	–	–	–	–
Firm age	2.006	0.616	0.172^***^	0.150^**^	0.128^*^	0.160^***^	–	–	–	–	–	–	–
Firm size	1.615	0.739	0.005	0.037	0.081	0.108	0.310^*^	–	–	–	–	–	–
Environmental dynamics	0.875	0.331	−0.034	−0.007	−0.021	0.024	0.301^*^	0.191	–	–	–	–	–
Competition intensity	0.872	0.335	0.007	−0.015	−0.059	0.002	0.190^*^	0.120^*^	0.202^*^	–	–	–	–
Market performance	3.665	1.098	0.520^***^	0.302^**^	0.276^**^	0.264^*^	0.126^*^	0.107	0.059	0.041	–	–	–
Financial performance	3.679	0.993	−0.213^***^	0.011	−0.092	−0.074	0.012	0.053	−0.044	−0.013	−0.133^*^	–	–
Individualistic identity orientation	1.676	0.946	−0.280^**^	−0.029	−0.106	−0.002	−0.011	0.049	−0.030	0.003	0.071	−0.054	–
Relational identity orientation	1.891	0.943	0.170^**^	−0.009	0.065	0.082	0.003	−0.038	0.010	−0.006	−0.142^**^	0.043	−0.356^***^

^***^ means *p* < 0.001, ^**^ means *p* < 0.01, ^*^ means *p* < 0.05.

At the same time, we also carried out correlation analysis on all variables to measure the degree of correlation between two variables. According to correlation coefficient shown in Table 2, we can know that correlation coefficients of servitization and normative pressure (*β* = 0.233, *p* < 0.001), servitization and mimetic pressure (*β* = 0.300, *p* < 0.001), and servitization and coercive pressure (*β* = 0.248, *p* < 0.001) are positive and significant, respectively. Meanwhile, the correlation coefficient between servitization and market performance is positive and significant (*β* = 0.520, *p* < 0.001), while the correlation coefficient between servitization and financial performance is negative and significant (*β* = −0.213, *p* < 0.001).

### Measurement Assessment: Reliability, Validity, Sample and Method Variance

To test reliability of the multi-item constructs, we used Cronbach’s Alpha, the most commonly used reliability index developed by Likert scale (Table 3). The reliability analysis is carried out for variables with multiple measurement items, such as normative pressure, mimetic pressure, coercive pressure, servitization, market performance, and financial performance in the research model. The specific reliability coefficients are shown in Table 4. The reliability coefficient Cronbach’s Alpha of all variables is greater than 0.80, indicating that the scale adopted in this study has good internal reliability.

**Table 3:** Construct measurement and confirmatory factor analysis results.

Constructs	Measurements	Standardized factor loadings	Cronbach’*α*
Servitization	Preventive maintenance, repair, maintenance services	0.847	0.929
	Technology upgrade services	0.735	
	Health monitoring service	0.790	
	Commissioning and installation service	0.698	
	Technical transformation service	0.749	
	Technical support for new product development	0.689	
	Business process optimization recommendations	0.727	
	Customer information service	0.747	
	Professional technical support related to production system design and transformation	0.743	
	New project establishment related reference suggestion	0.800	
Normative pressure	Many vendors have adopted servitization strategies	0.808	0.843
	Many of the partners have adopted servitization strategies	0.690	
	Many other companies in the industry have adopted the servitization strategy	0.914	
Mimetic pressure	Many of our competitors have adopted servitization strategies	0.804	0.835
	Competitors have realized differentiated competitive advantages after adopting servitization strategy	0.674	
	Competitors gain more customer loyalty after adopting servitization strategy	0.920	
Coercive pressure	Customers want the company to provide more services	0.830	0.857
	You may not be able to retain existing customers if you do not service them	0.722	
	Your company has a large number of customers in this field	0.906	
Financial performance	Servitization strategy will bring higher profits to your company	0.817	0.893
	Higher profit margins on sales	0.854	
	Higher return on investment	0.906	
Market performance	Servitization strategy will bring higher customer satisfaction to your company	0.812	0.878
	Higher customer loyalty	0.798	
	Increase in market share	0.913	

**Table 4:** Reliability analysis and Validity analysis on all variables used in this study.

Variables	Reliability value	AVE	AVE square root
Servitization	0.929	0.738	0.859
Normative pressure	0.843	0.649	0.806
Mimetic pressure	0.835	0.568	0.754
Coercive pressure	0.857	0.655	0.809
Financial performance	0.894	0.677	0.823
Market performance	0.886	0.712	0.844

We used confirmatory factor analysis and the average extraction variance (AVE) (Paulraj et al., 2008) to estimate the aggregate validity and discriminant validity. It is generally believed that the aggregate validity of this scale is acceptable when all standardized factor loading are greater than 0.50 and AVE is greater than 0.50. If the square root of AVE is greater than the correlation coefficient between latent variables, it proves that our measurement of this latent variable can be distinguished from other scales.

According to Table 4, the standardized factor loading are all greater than 0.674, reliability values are all greater than 0.80, and AVE values of each latent variable are all greater than 0.50, indicating that the scale has a good aggregate validity. Moreover, according to Table 4, the square root of AVE of each latent variable is greater than the maximum correlation coefficient between latent variables (0.520), which proves that the discriminative validity of this scale is good.

We compared the early and late responses to all variables using a *T*-test to assess the potential non-response bias (Podsakoff and Organ, 1986). No significant differences were found in non-response bias, indicating it is not an issue in this study. Since there was a single respondent per company, we checked the potential for common method bias using Harman’s one-factor test (Armstrong and Overton, 1977). The exploratory factor analysis of the multiple-item scales was conducted and resulted in a five-factor solution that accounted for 77.2% of the total variance. Factor 1 accounted for 22.1% of the variance, revealing that common method bias was not a problem in this study.

## Results

### Institutional Pressure on Manufacturers’ Servitization Strategy

Multiple linear regression is used to test the relationship between institutional pressure and manufacturer’s servitization strategy, that is, to verify the positive influence of normative pressure, mimetic pressure and coercive pressure on manufacturer’s servitization strategy. To be specific, after controlling the influence of variables such as enterprise age, enterprise scale, nature of enterprise ownership, environmental dynamics and competition intensity, we test whether the three kinds of institutional pressures have significant influence on manufacturers’ servitization strategy. The specific results are shown in Table 5.

**Table 5:** Regression analysis of institutional pressure on servitization strategy of manufacturers.

		Manufacturer servitization strategy			
		Model 1	Model 2	Model 3	Model 4
Constant		3.330^***^ (3.649)	2.737^**^ (3.006)	2.545^**^ (2.867)	3.118^***^ (3.500)
Independent variables	Normative pressure	–	0.169^***^ (3.580)	–	–
	Mimetic pressure	–	–	0.225^***^ (5.244)	–
	Coercive pressure	–	–	–	0.182^***^ (4.125)
Control variables	Firm age	0.390^***^ (3.651)	0.326^**^ (3.070)	0.335^**^ (3.251)	0.338^**^ (3.223)
	Firm size	−0.213^*^ (−2.375)	−0.193^*^ (−2.181)	−0.214^*^ (−2.482)	−0.218^*^ (−2.491)
	Environmental dynamics	0.055 (0.250)	0.074 (0.345)	0.065 (0.310)	0.059 (0.277)
	Competition intensity	−0.051 (−0.246)	−0.047 (−0.229)	0.001 (0.006)	−0.044 (−0.216)
	Nature of Company 1	−0.035 (−0.036)	0.023 (0.025)	0.175 (0.189)	−0.490 (−0.291)
	Nature of Company 2	−0.203 (−0.227)	−0.119 (−0.135)	−0.135 (−0.157)	−0.637 (−0.577)
	Nature of Company 3	−0.181 (−0.204)	−0.111 (−0.127)	−0.084 (−0.098)	0.059 (−0.562)
	Nature of Company 4	−0.331 (−0.370)	−0.243 (−0.276)	−0.262 (−0.305)	−0.044 (−0.727)
*R* ^2^		0.049	0.088	0.129	0.100
Adjusted *R*^2^		0.024	0.061	0.103	0.073
*R*^2^ change		–	0.039	0.080	0.051
*F*-statistic		1.966	3.240	4.955	3.73

^***^ means *p* < 0.001, ^**^ means *p* < 0.01, ^*^ means *p* < 0.05. The variables are displayed as coefficient (*T* value).

Regression analysis shows that the coefficient of normative pressure and manufacturers’ servitization strategy is positive and significant (*β* = 0.169, *p* < 0.001), indicating that there is a positive relationship between normative pressure and manufacturers’ servitization strategy. In other words, the stronger the perceived normative pressure is, the higher the probability of enterprises to adopt servitization strategy will be. Therefore, the hypothesis H1 of this study is supported. The coefficient of mimetic pressure and manufacturers’ servitization strategy is positive and significant (*β* = 0.225, *p* < 0.001), indicating a positive relationship between mimetic pressure and manufacturers’ servitization strategy. In other words, the stronger the mimetic pressure perceived by enterprises, the higher the probability of enterprises’ adopting servitization strategy will be. Therefore, the hypothesis H2 in this study is supported. The coefficient of coercive pressure and manufacturers’ servitization strategy is positive and significant (*β* = 0.182, *p* < 0.001), indicating that there is a positive relationship between coercive pressure and manufacturers’ servitization strategy. In other words, the stronger the perceived forced pressure is, the higher the probability of enterprises’ adopting servitization strategy will be. Therefore, the hypothesis H3 in this paper is supported.

### Moderating Effect of Individualistic Identity Orientation and Relational Identity Orientation

According to model 6, the regression coefficients of the interaction term between normative pressure and Individualistic identity orientation and manufacturers’ servitization strategy were negative and significant (*β* = − 0.087, *p* < 0.01; Table 6). The results show that individualistic identity orientation weakens the relationship between normative pressure and manufacturers’ servitization strategy, and the hypothesis H4a is supported. According to model 7, the regression coefficient of the interaction term between mimetic pressure and individualistic identity orientation and manufacturers’ servitization strategy was negative and significant (*β* = −0.066, *p* < 0.05). The results show that individualistic identity orientation weakens the relationship between mimetic pressure and manufacturer’s servitization strategy, and the hypothesis H4b is supported. According to model 8, the regression coefficient of the interaction term between coercive pressure and individualistic identity oriented was negative but not significant (*β* = −0.051, *p* > 0.1). The results show that individualistic identity orientation does not affect the relationship between coercive pressure and manufacturer’s servitization strategy, and the hypothesis H4c is not supported.

**Table 6:** The moderating effect of individualistic identity orientation.

		Manufacturer servitization strategy			
		Model 5	Model 6	Model 7	Model 8
Interactive items	Normative pressure × Individualistic identity orientation	–	−0.087^**^ (−2.837)	–	–
	Mimetic pressure × Individualistic identity orientation	–	–	−0.066^*^ (−2.258)	–
	Coercive pressure × Individualistic identity orientation	–	–	–	−0.051 (−1.072)
Independent variables	Normative pressure	0.122^**^ (2.766)	0.119^**^ (2.721)	0.117^**^ (2.658)	0.118^**^ (2.665)
	Mimetic pressure	0.229^***^ (5.659)	0.219^***^ (5.446)	0.231^***^ (5.742)	0.216^***^ (5.273)
	Coercive pressure	0.169^***^ (4.073)	0.162^***^ (3.943)	0.153^***^ (3.668)	0.164^***^ (3.968)
Moderating variable	Individualistic identity orientation	−0.086^*^ (−2.561)	−0.085^*^ (−2.544)	−0.080^*^ (−2.424)	−0.093^**^ (−2.828)
Control variables	Firm age	0.222^*^ (2.253)	0.214^*^ (2.202)	0.203^*^ (2.072)	0.232 ^*^ (2.356)
	Firm size	−0.207^*^ (−2.552)	−0.211^**^ (−2.641)	−0.202^*^ (−2.516)	−0.211^**^ (−2.609)
	Environmental dynamics	0.078 (0.393)	0.104 (0.530)	0.141 (0.709)	0.085 (0.433)
	Competitive intensity	0.016 (0.086)	0.003 (0.014)	−0.014 (−0.076)	−0.002 (−0.009)
	Nature of Company 1	0.274 (0.314)	0.239 (0.277)	0.170 (0.195)	0.491 (0.558)
	Nature of Company 2	−0.095 (−0.116)	−0.165 (−0.206)	−0.117 (−0.145)	0.062 (0.077)
	Nature of Company 3	−0.053 (−0.066)	−0.127 (−0.159)	−0.080 (−0.100)	0.109 (0.134)
	Nature of Company 4	−0.229 (−0.282)	−0.339 (−0.423)	−0.282 (−0.349)	−0.064 (−0.079)
*R* ^2^		0.241	0.261	0.253	0.248
Adjusted *R*^2^		0.210	0.228	0.221	0.215
*R*^2^ change		0.062	0.020	0.012	0.007
*F*-statistic		7.890	8.074	7.775	7.552

^***^ means *p* < 0.001, ^**^ means *p* < 0.01, ^*^ means *p* < 0.05. The variables are displayed as coefficient (*T* value).

According to model 10 (Table 7), the regression coefficient of the interaction term between normative pressure and relational identity oriented and manufacturers’ servitization strategy is positive but not significant (*β* = 0.029, p > 0.1). The results show that relational identity orientation does not affect the relationship between normative pressure and the manufacturers’ servitization strategy, and the hypothesis H5a is not supported. According to model 11, the regression coefficient of the interaction term between mimetic pressure and relational identity orientation and manufacturers’ servitization strategy was positive and significant (*β* = 0.095, *p* < 0.01). The results show that relational identity orientation positively moderates the relationship between mimetic pressure and the manufacturers’ servitization strategy, and the hypothesis H5b is supported. According to model 12, he regression coefficient of the interaction term between coercive pressure and relational identity orientation was positive and significant (*β* = 0.095, *p* < 0.01). The results show that relational identity orientation positively moderates the relationship between coercive pressure and the manufacturers’ servitization strategy, and the hypothesis H5c has been verified.

**Table 7:** The moderating effect of relational identity orientation.

		Manufacturer servitization strategy			
		Model 9	Model 10	Model 11	Model 12
Interactive items	Normative pressure × Relational identity orientation	–	0.029 (0.953)	–	–
	Mimetic pressure × Relational identity orientation	–	–	0.095^**^ (3.246)	–
	Coercive pressure × Relational identity orientation	–	–	–	0.095^**^ (3.210)
Independent variables	Normative pressure	0.120^**^ (2.682)	0.120^**^ (2.681)	0.104^*^ (2.343)	0.102^*^ (2.292)
	Mimetic pressure	0.215^***^ (5.238)	0.209^***^ (5.050)	0.216^***^ (5.344)	0.183^***^ (4.411)
	Coercive pressure	0.168^***^ (3.980)	0.163^***^ (3.835)	0.137^**^ (3.212)	0.173^***^ (4.164)
Adjust the variable	Relational identity orientation	0.132^***^ (4.068)	0.131^***^ (4.087)	0.128^**^ (3.969)	0.129^***^ (3.993)
Control variables	Firm age	0.215^*^ (2.142)	0.208^*^ (2.065)	0.220^*^ (2.225)	0.216^*^ (2.177)
	Firm size	−0.187^*^ (−2.265)	−0.185^*^ (−2.240)	−0.195^*^ (−2.395)	−0.196^*^ (−2.406)
	Environmental dynamics	0.061 (0.304)	0.070 (0.348)	0.137 (0.687)	0.060 (0.300)
	Competitive intensity	−0.003 (−0.018)	−0.007 (−0.036)	−0.021 (−0.110)	−0.043 (−0.224)
	Nature of Company 1	−0.012 (−0.013)	−0.018 (−0.021)	0.005 (0.005)	0.114 (0.131)
	Nature of Company 2	−0.269 (−0.326)	−0.274 (−0.333)	−0.250 (−0.309)	−0.123 (−0.152)
	Nature of Company 3	−0.239 (−0.292)	−0.249 (−0.303)	−0.216 (−0.267)	−0.089 (−0.110)
	Nature of Company 4	−0.426 (−0.518)	−0.446 (−0.541)	−0.417 (−0.515)	−0.289 (−0.357)
*R* ^2^		0.216	0.218	0.241	0.242
Adjusted *R*^2^		0.184	0.184	0.209	0.209
*R*^2^ change		0.037	0.002	0.025	0.026
*F*-statistic		6.852	6.393	7.337	7.315

^***^ means *p* < 0.001, ^**^ means *p* < 0.01, ^*^ means *p* < 0.05. The variables are displayed as coefficient (*T* value).

### Manufacturers’ Servitization Strategy and Performance

The regression analysis of manufacturers’ servitization strategy on financial performance shows that the coefficient of manufacturers’ servitization strategy is negative and significant (*β* = −0.235, *p* < 0.001; Table 8). It shows that the manufacturers’ servitization strategy has a negative impact on financial performance, that is, the higher the level of manufacturers’ servitization strategy, the worse the financial performance. The regression analysis of manufacturer’s servitization strategy on market performance shows that the coefficient of the manufacturer’s servitization strategy is positive and significant (*β* = 0.653, p < 0.001). It shows that the manufacturers’ servitization strategy has a positive impact on market performance, that is, the higher the level of the manufacturers’ servitization strategy, the better the market performance.

**Table 8:** The effect of manufacturer’s servitization strategy on enterprise performance.

		Financial performance	Market performance
Constant	–	5.056^***^ (4.960)	1.835 (1.863)
Independent variables	Servitization	−0.235^***^ (−3.712)	0.653^***^ (10.650)
Control variables	Firm age	0.045 (0.383)	−0.109 (−0.968)
	Firm size	0.072 (0.717)	0.172 (1.784)
	Nature of enterprise 1	−0.733 (−0.689)	−0.588 (−0.572)
	Nature of enterprise 2	−0.808 (−0.818)	−0.427 (−0.448)
	Nature of enterprise 3	−0.693 (−0.705)	−0.646 (−0.680)
	Nature of enterprise 4	−0.794 (−0.803)	−0.458 (−0.480)
*R* ^2^		0.050	0.288
Adjusted *R*^2^		0.028	0.272
*R*^2^ change		–	–
*F*-statistic		2.302	17.58

^***^ means p < 0.001. The variables are displayed as coefficient (T value).

### The impact of Manufacturers’ Servitization Strategy On Performance based On Institutional Pressure Prediction

In order to further verify whether institutional pressure is the cause of the phenomenon of “service paradox,” we adopt the method presented in previous study (Castellaneta et al., 2018). First of all, we use normative pressure, mimetic pressure, coercive pressure, enterprise age and number of employees to conduct regression analysis on manufacturers’ servitization strategy. The results are shown in Table 9. Normative pressure, mimetic pressure, coercive pressure, enterprise age and number of employees all have significant influences on manufacturers’ servitization strategy.

**Table 9:** Regression analysis of institutional pressures on servitization strategy of manufacturers.

		Servitization strategy
Constant	–	1.676^***^ (6.644)
Independent variables	Normative pressure	0.126^**^ (2.819)
	Mimetic pressure	0.212^***^ (5.179)
	Coercive pressure	0.155^***^ (3.683)
Control variables	Firm age	0.254^**^ (2.646)
	Firm size	−0.188^*^ (−2.393)
*R* ^2^		0.191
Adjusted *R*^2^		0.177
*R*^2^ change		–
*F*-statistic		14.42

^***^ means *p* < 0.001, ^**^ means *p* < 0.01, ^*^ means *p* < 0.05. The variables are displayed as coefficient (*T* value).

Then, we obtain the regression equation of the manufacturer’s servitization strategy according to the regression coefficient in the above model. The servitization strategy level of institutional pressure prediction is calculated, and then the difference absolute value between the predicted servitization strategy level and the actual servitization strategy level is obtained.

MS represents the level of servitization strategy actually adopted by the enterprise, while point MS’ represents the level of servitization strategy predicted by the institutional pressures. ABS (MS–MS’) represents the absolute distance between MS and MS’, that is, the absolute value of the difference between the servitization strategy level actually adopted by the enterprise and the servitization strategy level predicted by the institutional pressure. When the distance between point MS and point MS’ is larger, the ABS (MS–MS’) value will be larger, which means that the servitization strategy level actually adopted by the enterprise will deviate more from the servitization strategy level predicted by the institutional pressure, that is, the enterprise does not adopt servitization in accordance with the institutional pressures. On the contrary, the smaller the distance between point MS and point MS’ is, the smaller the ABS (MS–MS’) value will be, which means that the servitization strategy level actually adopted by enterprises will be more in line with the servitization strategy level predicted by the institutional pressure, that is, enterprises will adopt servitization under institutional pressures.

Absolute residual ABS (MS–MS’) was used to conduct regression analysis on financial performance and market performance, respectively. The specific analysis results are shown in Table 10 below. The regression coefficient of absolute residual ABS (MS–MS’) on financial performance is positive and significant (*β* = 0.196, *p* = 0.07), indicating that manufacturer’s servitization strategy in accordance with the prediction of institutional pressure will lead to worse financial performance compared with manufacturer’s servitization strategy not in accordance with the prediction of institutional pressure. As we have previously proved that the manufacturer’s servitization strategy has a negative impact on financial performance, it also indicates that the manufacturer’s servitization strategy under institutional pressure will lead to negative financial performance, which also leads to the emergence of the “service paradox” phenomenon. The regression coefficient of residual ABS (MS–MS’) on market performance is negative but not significant (*β* = −0.165, *p* > 0.1), which cannot explain whether the servitization strategy brought by institutional pressure will lead to better or worse market performance.

**Table 10:** Regression analysis of servitization level residuals on enterprise performance.

		Financial performance	Market performance
Independent variables	ABS (MS – MS′)	0.196# (1.768)	−0.165 (−1.352)
Control variables	Firm age	−0.018 (−0.154)	0.137 (1.070)
	Firm size	0.064 (0.650)	0.095 (0.884)
*R* ^2^		0.014	0.022
Adjusted *R*^2^		0.004	0.012
*R*^2^ change		–	–
*F*-statistic		1.423	2.285

*# means *p* < 0.1. The variables are displayed as coefficient (*T* value)*.

## Discussion

### Main Conclusions

Based on institutional theory and related theories of organizational identity orientation, this study develops and tests a service-oriented transformation model for manufacturing enterprises under institutional pressure. Using sample data from 312 enterprises in 12 manufacturing sub-sectors in China, this study empirically tests the assumptions and the theoretical model proposed in this study and the following main research conclusions are drawn (Table 11).

**Table 11:** Summary of the hypotheses included in this study.

Hypotheses	Description	Whether support or not
H1	The perceived normative pressure by the manufacturer positively affects the manufacturer to adopt servitization strategy	Yes
H2	The mimetic pressure perceived by the manufacturer positively affects the servitization strategy of the manufacturer.	Yes
H3	The coercive pressure perceived by the manufacturer positively affects the servitization strategy of the manufacturer.	Yes
H4a	Individualistic identity orientation negatively moderates the impact of normative pressure on manufacturer servitization.	Yes
H4b	Individualistic identity orientation negatively moderates the impact of mimetic pressure on manufacturer servitization.	Yes
H4c	Individualistic identity orientation positively moderates the impact of coercive pressure on manufacturer servitization.	No
H5a	Relationship identity orientation positively moderates the impact of normative pressure on manufacturer servitization.	No
H5b	Relational identity orientation positively moderates the impact of mimetic pressure on manufacturer servitization.	Yes
H5c	Relational identity orientation negatively moderates the impact of coercive pressure on manufacturer servitization.	Yes
H6	The servitization strategy of manufacturers positively affects the marketing performance of enterprises.	Yes
H7	The servitization strategy of manufacturers negatively affects the financial performance of enterprises.	Yes

First, this study verifies that institutional pressure (normative, mimetic, and coercive pressures) will significantly and positively affect the manufacturer’s servitization strategy. The results show that the institutional pressure perceived by enterprises increase the willingness of manufactures to adopt a servitization strategy. This also shows that the adoption of servitization strategies of enterprises is not only driven by internal economic benefits, but also affected by institutional pressure from stakeholders in the external environment.

Second, this study verifies that manufacturing firms with individualistic identity orientation tend to adopt less servitization strategies when faced with normative and imitative pressures. When faced with coercive pressure, the moderating effect of individualistic identity orientation is not significant, which may be related to the industry in which the company is located. If the customers in the industry in which the company is located are relatively stable and change little, companies with individualistic identity orientation may respond to institutional pressure, as the business will suffer direct economic losses if the customer’s service needs are not met. When faced with mimetic pressure and coercive pressure, manufacturing enterprises with relational identity orientation tend to adopt more servitization strategies. When faced with normative pressure, the moderating effect of relational identity orientation was not significant.

Third, this study divides corporate performance into financial performance and market performance, and examines the impact of manufacturing servitization strategies on these two types of performance. The results show that the manufacturing servitization strategy negatively affects financial performance, that is, servitization cannot bring good financial performance. This is most likely because Chinese manufacturing companies started their service-oriented transformation later than those in developed countries. Therefore, it is very likely that they are still in the stage of massive investment in services and have not yet achieved economies of scale in services (Kastalli et al., 2013). The results also show that the servitization strategy of manufacturing has a positive impact on market performance, that is, servitization can bring good market performance to manufacturing companies. By classifying firm performance, we have a clearer understanding of the relationship between manufacturing servitization strategies and performance, and confirm the phenomenon of the “servitization paradox” (Visnjic et al., 2012). At the same time, we use residual regression to test that the manufacturer’s servitization strategy predicted by institutional pressure will lead to poor financial performance, and also confirm that this “servitization paradox” phenomenon is likely due to the fact that companies succumb to institutional pressure and adopt a servitization strategy.

### Theoretical Implications

First, based on institutional theory, the impact of institutional pressure on the servitization strategy of manufacturing industry is tested for the first time and the application of institutional pressure is promoted. Previous research on the impact of institutional pressure on enterprises has mainly focused on corporate social responsibility behavior, ethical behavior, innovation adoption (Teo et al., 2003; Liang et al., 2007; Liu et al., 2010; Martin et al., 2011). This study also expands the research on the driving factors of the existing manufacturers’ servitization strategy. Most of the existing researches focus on the reasons for the manufacturer’s servitization strategy from the perspective of the internal economy of the enterprise. While considering economic benefits, enterprises will inevitably be affected by institutional pressure. In order to obtain rationality and status in the network, enterprises may succumb to institutional pressure to make strategic decisions. Therefore, institutional pressure is also a possible driving factor for enterprises to make servitization strategies.

Second, this study examines the boundary conditions for the impact of institutional pressure on firm strategic behavior from a new perspective. In the past, the research on the effect of institutional pressure on enterprise innovation decision-making was chaotic. Some studies found the effect of coercive pressure to be significant (Teo et al., 2003), while others found it to be insignificant (Liang et al., 2007). In view of this, more research is needed to explore the potential moderating variables in the process of enterprises responding to institutional pressure. Currently, only Li and Ding (2013) have studied that companies with strong capabilities will reduce their dependence on the external environment and reduce the imitation behavior of their peers’ corporate strategies (Li and Ding, 2013). And Liu et al. (2010) combined institutional theory and organizational culture theory to study the moderating effect of organizational culture (Liu et al., 2010). It can be seen that there is a lack of boundary conditions research on the impact of institutional pressure on corporate strategic behavior. The introduction of the concept of organizational identity orientation in this study helps to increase the understanding of the process of corporate response to institutional pressure.

Third, we have more understandings of the relationship between servitization strategy and performance by classifying corporate performance into market performance and financial performance for the empirical research, While studies have demonstrated the benefits of service on the performance of the product itself and in creating customer value, the impact of this innovation on manufacturer performance is unclear, and past studies about the impact of servitization strategy on manufacturers’ performance have yielded conflicting results (Fang et al., 2008; Neely, 2008; Visnjic et al., 2012; Suarez et al., 2013; Marjanovic et al., 2020). Although some of these studies have classified firm performance, few studies have confirmed the different effects of servitization strategies on them. This study confirms that manufacturing servitization strategies have a positive impact on market performance and has a negative effect on financial performance, demonstrating the phenomenon of the “servitization paradox.”

Fourth, this study adopts a new confirmatory method using the servitization strategy predicted by institutional pressure to do regression analysis on the performance of the enterprise (Castellaneta et al., 2018). It is confirmed that if the enterprise succumbs to the institutional pressure and implements the servitization strategy, it will produce poor financial performance, which also shows that the institutional pressure is one of the causes in the phenomenon of “servitization paradox.” Therefore, this study proposes a new explanation for the “servitization paradox” based on institutional theory, expanding the understanding of the phenomenon of “servitization paradox.”

### Practical Implications

First, in the process of pursuing internal economic benefits, enterprises should consider the influence from the external institutional environment. The empirical research of this study shows that the institutional pressure from suppliers, partners, competitors, and target customers perceived by enterprises will affect the decision-making of servitization strategies of manufacturing enterprises. Due to the uncertainty of the performance of manufacturers’ servitization strategies and the risks existing in the implementation of new strategies, study on the impact of institutional pressure can help manufacturing enterprises to better understand the strategic choices of enterprises under uncertainty and risks.

Second, we can gain a clearer understanding of how firms strategically respond to institutional pressures by demonstrating the moderating effect of organizational identity orientation between institutional pressures and servitization strategies. This study confirms that firms with individualistic identity orientation will weaken the influence of institutional pressure on servitization strategies, while firms with relational identity orientation will strengthen the influence of institutional pressures on servitization strategies. This provides guidance for companies to respond to external institutional pressures. Excessive submission to institutional pressures may lead to poor performance results, and it is necessary to flexibly combine their own resource endowments to make strategic choices.

Third, this study demonstrates the different effects of manufacturing servitization strategies on market performance and financial performance, and confirms the many benefits that manufacturing servitization strategies mentioned in past research will bring to enterprises, such as customer satisfaction and differentiated competitive advantages, also confirms that the servitization strategy may not bring good financial performance, because services require investment and service benefits are long-term and persistent characteristics, and service investment may not necessarily increase the profit margins and ROI (Return On Investment) of enterprise in a short period of time. This provides a reference for enterprises to choose a servitization strategy and enterprises should choose a servitization strategy according to the goals they want to achieve.

Fourth, this samples in this study come from the Chinese market, and the conclusions obtained are applicable to Chinese manufacturing enterprises. Chinese culture provides fertile ground for the study of institutional pressures because of collectivistic qualities, high power distance, and emphasis on guanxi and face. In collectivist cultures, group interests predominate. Members of a collective culture are likely to subordinate individual goals to the group, putting the collective interests ahead of their own, which sets the stage for institutional pressure. Moreover, the emphasis on guanxi and face makes it easy to observe the effects of institutional pressure on Chinese firms. For example, a company might adopt an innovation to avoid losing face because of good relationships with other members. From this perspective, Chinese culture serves as a lens to more clearly describe the role of institutional pressures in Chinese business practices. According to the research results of this study, we provide useful guidance for Chinese manufacturing enterprises to break the bottleneck of development.

### Limitations and Directions for Future Research

There are some unavoidable limitations of this paper. This study mainly starts from the driving factors, and tries to explain the phenomenon of “servitization paradox” through the influence of institutional pressure on the adoption of servitization strategies of manufacturers. However, to provide more theoretical explanations for the “servitization paradox” phenomenon, more empirical research on the moderator variables of the relationship between manufacturers’ servitization strategies and firm performance is needed. Although past studies have proposed many moderating variables, such as corporate resources, organizational capabilities and corporate cultural aspirations, core competencies, delivery capabilities, and service capabilities (Mathieu, 2001; Oliva and Kallenberg, 2003; Baines et al., 2009; Martinez et al., 2010). However, there are still few empirical studies, and future research can consider using empirical studies to confirm whether these moderator variables significantly affect the relationship between servitization strategy and performance. Second, although the author has adopted a scientific way to design and collect data, there are still limitations in research samples and data. In the process of data collection, it is unavoidable to be limited by objective conditions, which leads to a certain degree of defects in the sampling scope and data collection of this paper. Therefore, follow-up research can consider improving these aspects and improve the generality of the research.

## Data Availability Statement

The data that supports the findings of this study are available from the corresponding author, upon reasonable request.

## Author Contributions

HuW and HaW: conceptualization. HuW, XL, HaW, and CH: methodology. HuW and XL: formal analysis and investigation. HuW: writing – original draft preparation. HuW, XL, HaW, and CH: writing – review and editing. HuW: funding acquisition. XL, HaW, and CH: resources. HaW: supervision. All authors contributed to the article and approved the submitted version.

## Funding

This study was supported by the Philosophy and Social Science Research Project in Colleges and Universities of Hubei Province (Grant number: 21Q123).

## Conflict of Interest

The authors declare that the research was conducted in the absence of any commercial or financial relationships that could be construed as a potential conflict of interest.

## Publisher’s Note

All claims expressed in this article are solely those of the authors and do not necessarily represent those of their affiliated organizations, or those of the publisher, the editors and the reviewers. Any product that may be evaluated in this article, or claim that may be made by its manufacturer, is not guaranteed or endorsed by the publisher.
